# Multi-generation study of heavy ion beam-induced mutations and agronomic trait variations to accelerate rice breeding

**DOI:** 10.3389/fpls.2023.1213807

**Published:** 2023-06-21

**Authors:** Weibin Ren, He Wang, Yan Du, Yan Li, Zhuo Feng, Xinhui Zhou, Guisen Kang, Qingyao Shu, Tao Guo, Huijun Guo, Lixia Yu, Wenjie Jin, Fu Yang, Jingpeng Li, Jianzhong Ma, Wenjian Li, Chaoli Xu, Xia Chen, Xiao Liu, Chenan Yang, Luxiang Liu, Libin Zhou

**Affiliations:** ^1^Biophysics Group, Biomedical Center, Institute of Modern Physics, Chinese Academy of Sciences, Lanzhou, China; ^2^University of Chinese Academy of Sciences, Beijing, China; ^3^State Key Laboratory of Crop Gene Exploration and Utilization in Southwest China, Sichuan Agricultural University, Chengdu, China; ^4^State Key Laboratory of Rice Biology, Institute of Biotechnology, Zhejiang University, Hangzhou, China; ^5^National Engineering Research Center of Plant Space Breeding, College of Agriculture, South China Agricultural University, Guangzhou, China; ^6^National Key Facility for Crop Gene Resources and Genetic Improvement, National Center of Space Mutagenesis for Crop Improvement, Institute of Crop Sciences, Chinese Academy of Agricultural Sciences, Beijing, China; ^7^Northeast Institute of Geography and Agroecology, Chinese Academy of Sciences, Changchun, China; ^8^School of Life Science and Engineering, Lanzhou University of Technology, Lanzhou, China; ^9^College of Life Science and Technology, Gansu Agricultural University, Lanzhou, China

**Keywords:** heavy ion beam, mutagenesis, rice (*Oryza sativa*), whole-genome sequencing, mutation characteristics, mutant screening

## Abstract

Heavy ion beam (HIB) is an effective physical mutagen that has been widely used in plant mutational breeding. Systemic knowledge of the effects caused by different HIB doses at developmental and genomic levels will facilitate efficient breeding for crops. Here we examined the effects of HIB systematically. Kitaake rice seeds were irradiated by ten doses of carbon ion beams (CIB, 25 – 300 Gy), which is the most widely used HIB. We initially examined the growth, development and photosynthetic parameters of the M_1_ population and found that doses exceeding 125 Gy caused significant physiological damages to rice. Subsequently, we analyzed the genomic variations in 179 M_2_ individuals from six treatments (25 – 150 Gy) *via* whole-genome sequencing (WGS). The mutation rate peaks at 100 Gy (2.66×10^-7^/bp). Importantly, we found that mutations shared among different panicles of the same M_1_ individual are at low ratios, validating the hypothesis that different panicles may be derived from different progenitor cells. Furthermore, we isolated 129 mutants with distinct phenotypic variations, including changes in agronomic traits, from 11,720 M_2_ plants, accounting for a 1.1% mutation rate. Among them, about 50% possess stable inheritance in M_3_. WGS data of 11 stable M_4_ mutants, including three lines with higher yields, reveal their genomic mutational profiles and candidate genes. Our results demonstrate that HIB is an effective tool that facilitates breeding, that the optimal dose range for rice is 67 – 90% median lethal dose (LD_50_), and that the mutants isolated here can be further used for functional genomic research, genetic analysis, and breeding.

## 1 Introduction

Food security has increasingly become a prominent issue with the rapid increase in the population and climate change. Rice is one of the most important crops in the world feeding over half of the world’s population (Gross and Zhao, 2014). Thus, there is a great need in rice breeding to meet the demand. Mutations not only directly provide rich resources for breeding, but also genetic resources for functional genomics study, molecular design, and other technologies. Physical mutagenesis has played an important role in the construction of mutant populations for a long time. Currently, commonly used physical mutagens include X-rays, γ-rays, fast neutron (FN), and heavy-ion beams (HIB). HIB is a unique physical mutagen, whose role in mutant creation is also gradually favored by breeders.

HIB is a kind of ionizing radiation generated from the acceleration of heavy ions by a large-scale particle accelerator. Compared with traditional physical mutagens like X-rays and γ- rays, HIB has higher linear energy transfer (LET), meaning the energy of ionized particles deposited on its unit track is higher, leading to dense ionization in the sample (Kazama et al., 2017). Therefore, HIB can produce higher relative biological effects (RBE) and induce higher mutation frequency at lower radiation doses (Hirano et al., 2015). HIB has been widely used in plant mutation-mediated breeding and construction of mutant libraries. Researchers have used HIB to construct a large number of mutants and rich varieties in model plants(Tanaka et al., 2002; Kitamura et al., 2004; Magori et al., 2009; Du et al., 2018; Du et al., 2021), crops (Abe et al., 2002; Tanaka et al., 2002; Ishikawa et al., 2012; Liu et al., 2013; Zhang et al., 2022) and ornamental plants (Yamaguchi et al., 2009b; Hase et al., 2010; Matsumura et al., 2010). Various types of rice mutants have been generated by HIB irradiation, including plant height variants, leaf variants (Peng et al., 2019), low cadmium absorption(Ishikawa et al., 2012), grain variants (Li et al., 2017; Jiang et al., 2019), UV-B insensitive mutants (Takano et al., 2013), and salt-alkali tolerance (Zhang et al., 2022).

In general, the first process of mutational breeding is to irradiate plant seeds with suitable radiation doses. The irradiated seeds are planted for seed collection (M_1_ generation). Then, large-scale screening for mutants was usually carried out in the M_2_ generation, and the selected mutants were verified in higher generations. Since the irradiation treatment is the original step of the physical mutational breeding, the selection of suitable irradiation parameters is undoubtedly the groundwork for the mutant library construction. The input dose is an important factor in controlling the amount of genetic mutations (Ichida et al., 2019). Therefore, analyzing the relationship between the irradiation dose and its biological effects on the M_1_ generation, the genome variation spectrum in M_2,_ and generated phenotypic variations will greatly facilitate the formulation of the irradiation design and the whole breeding work. Currently, most of studies focus on one of the above aspects, lacking an integrated evaluation through multi-generational study to investigate multi-dimensional biological effects. For example, some studies have only given the irradiation dose range based on the survival rate and fertility of the M_1_ generation after irradiation and the phenotypic mutation rate in the M_2_ generation, lacking the whole genome-level knowledge (Yamaguchi et al., 2009a), or only based on the survival rate of the M_1_ generation after irradiation and the mutation frequency of different doses of carbon ion beams (CIB) in the exon region in the M_2_ generation. They often recommend an optimal radiation dose range but lack more comprehensive whole genome data in M_1_ to support it(Ichida et al., 2019).

Previous studies on HIB-induced mutation characteristics mainly focused on certain doses with supporting data only from high-generation (M_3_-M_6_) phenotypic mutants(Li et al., 2019; Yang et al., 2019; Zheng et al., 2020). There were few studies on the mutation characteristics induced by different doses of HIB using low-generation (M_1_ - M_2_) mutants without phenotypic bias. Therefore, the characteristics of mutations induced by different HIB doses in the whole genome of unbiased rice mutants remain unknown.

Kitaake, as a model rice variety, is small and easy to reproduce and has a short life cycle (about nine weeks) and high transformation efficiency. Multiple Kitaake mutation populations have been constructed, including RNAi populations (Wang et al., 2013), T-DNA insertional populations (Kim et al., 2013), and FN-mutagenized populations (Li et al., 2016; Li et al., 2017). Kitaake has also been used to study some aspects of rice biology, such as disease resistance (Liu et al., 2015; Zhou et al., 2018), microRNAs (Rodrigues et al., 2013), and the CRISPR-Cas9 technology (Xie et al., 2015). The reference genome data of Kitaake was released in 2019. Therefore, it is suitable for the study of radiobiological effects and mutation mechanisms (Li et al., 2016; Li et al., 2017; Jain et al., 2019). In this study, we first systematically studied the biological effects induced by ten CIB doses in the M_1_ generation. Next, we looked at the mutation characteristics induced by six CIB doses (25 – 150 Gy) in the M_2_ generation and at different panicles of a single M_1_ plant. We constructed mutant populations from eight CIB doses (25 – 200 Gy), selected mutants with agronomic traits in their M_2_ - M_4_ generations, and identified three mutants with higher grain yields. We also selected 11 stable mutants with various phenotypes at the M_4_ generation for re-sequencing and revealed their genomic mutational profiles of single base substitutions (SBSs) and small insertions and deletions (InDels). The candidate genes responsible for these mutant phenotypes were predicted.

## 2 Materials and methods

### 2.1 Materials and carbon ion beams radiation

Dry seeds of rice (*Oryza sativa* ssp Japonica, Kitaake), whose water content was adjusted to 12%, were used for mutagenic treatment. The samples were irradiated by CIB (^12^C^6+^, 967 MeV) generated by the Heavy Ion Research Facility in Lanzhou (HIRFL) at the Institute of Modern Physics, Chinese Academy of Sciences (IMP-CAS). The average LET value of CIB was 34 keV/μm. During CIB radiation, the seeds were placed in a 35 mm Petri dish, about 100 seeds were placed in each Petri dish, and the thickness of the seed sample was about 0.8 cm. The irradiation doses were set as 25, 50, 75, 100, 125, 150, 175, 200, 250 and 300 Gy, and the dose rate was approximately 60 Gy/min. Treatments for each dose had three replicates.

### 2.2 Determination of M_1_ seedling growth indexes

After CIB irradiation, 40 seeds were randomly selected from each petri dishes with three replicates at each dose. The selected seeds placed in a 10 ml centrifuge tube, added about 8 ml H_2_O_2_(1%) surface disinfection for 30 min, and then washed the seeds with distilled water 3 times. Then the seeds germinated in 90 mm Petri dishes covered by three layers of wet filter paper with deionized water. The petri dishes were placed in electro-heating standing-temperature cultivator at 28°C for germination. Seeds that developed both primary leaves and radicles were regarded as germinated. Germinated seeds were counted every day until the number did not increase, then the seed germination rates were calculated (Number of germinated seeds/total number of seeds × 100%).

Rice seedlings were transferred into 1-liter hydroponic boxes with 1× Kimura B Nutrient Solution and placed in a growth chamber. For the setting of temperature, light and humidity conditions, refer to the rice growing protocol of the Ronald lab (https://kitbase.ucdavis.edu/). Specifically, the fluorescent light was set to 14 hours with a light intensity of ~250 μmolm^-2^s^-2^, and the temperature was maintained at 26-28°C. During the 10 hours of darkness, the temperature was maintained at 24°C, and the relative humidity was set to 60%. Root length and shoot length were determined at the 9th day after culture. Seedling survival rates were measured on the 14th and 21st day, and no significant difference in survival rates was found, and statistical data from the 21st day were plotted.

When rice seedlings have grown for 30 days (at tillering stage), their chlorophyll contents and chlorophyll fluorescence parameters were measured. Chlorophyll content was determined as described by Porra et al. (Porra et al., 1989). First, 0.2 g of leaf samples, with the main vein removed and taken from the same position on the leaf, were placed in a mortar. A small amount of quartz sand and calcium carbonate powder were added, followed by 3 ml of 95% ethanol. The sample was homogenized and then an additional 5 ml of 95% ethanol was added. Grinding was continued until the leaf tissue residue was white. After resting for 5 minutes, the sample was transferred to a 15 ml centrifuge tube and rinsed with 95% ethanol 2-3 times until the residue was completely white. The resulting solution was transferred to a centrifuge tube and centrifuged at 4000 r/min for 10 minutes. The final volume was fixed at 15 ml, and 200 μL supernatant was used to determine the absorbance at 665 nm, 649 nm, and 470 nm using a TECAN infinite 200 microplate reader. All of the above processes were carried out in darkness. Three replicates with three technical replicates were carried out for each radiation dose group. For chlorophyll fluorescence parameters, the new leaves at the top of the main stem during the tillering stage were selected for measurement, including ETR (II) (absolute electron transfer rate of photosystems II), Y(II) (actual photosynthetic efficiency of photosystems II), Fv/Fm (maximal photochemical efficiency of photosystems II), and Y(NO) (quantum yield of unregulated energy dissipation in photosystems II), which were determined using a Pulse-Amplitude-Modulated Chlorophyll Fluorometer (Dual-PAM-100). Three replicates were carried out for each radiation dose group.

After seed germination and raising seedlings in the growth chamber for 14 days, the seedlings were transplanted outdoors in barrels (size: 23 × 21 × 18 cm), with three rice plants in each planting barrel, in Lanzhou (36°03′ N, 103°40′ E). At the mature stage, 12 plants were selected for each CIB dose for the measurement of their plant height, panicle number per plant, tiller number per plant, and seed setting rate. Each panicle of an individual M_1_ plant was separately harvested to construct the seed bank.

### 2.3 Whole genome re-sequencing and mapping of reads to reference genome

For re-sequencing, the M_2_ lines from irradiation with six CIB doses (25 – 150 Gy) were selected to study the induced mutation types. Ten M_1_ plants were selected for each irradiation dose; from each M_1_ plant, three panicles were generally selected (two panicles for a few individuals); one seed from each panicle was selected to grow an M_2_ plant. In M_4_ generation, 11 stable mutants with different phenotypes were selected for re-sequencing. Total genomic DNA was extracted from young leaves of M_2_ and M_4_ plants by the cetyltrimethylammonium bromide (CTAB) method. The integrity of DNA samples was assessed by 1% gel electrophoresis, and the OD_260/_OD_280_ ratio of DNA samples was measured with a TECAN infinite 200 microplate reader. High-quality DNA samples were used for genome resequencing. A total of 179 M_2_ samples (176 mutants and three controls) and 12 M_4_ samples (11 mutants and one control) were re-sequenced with an Illumina NovaSeq 6000 at Novogene Biotechnology Co., Ltd. Raw data were quality-controlled and filtered, and the obtained clean data were then mapped to the KitaakeX reference genome (Jain et al., 2019), using the Burrows-Wheeler-Alignment tool (Li and Durbin, 2009) and SAMtools (Li et al., 2009). The samples and sequence information of M_2_ and M_4_ generations are listed in **Supplementary Table 1**.

### 2.4 Genomic variant detection and mutation function annotation

In M_2_ generation, 13 samples, including ten M_2_ plants and three controls, were collected for each group. Individual sample from each panicle of the same M_1_ plant was kept separately to prevent shared mutations from being filtered. In M_4_ generation, 11 mutants and one control were used as a group for mutation detection. Detection of genomic variations was as described by Du et al. (2017) with minor modifications. SBSs and small InDels were screened using SAMtools (Li et al., 2009) and VarScan2 (v.3.9, http://varscan.sourceforge.net). Applying the “samtools mpileup -f ref.fa -q 10 samples_sorted.bam| java -jar VarScan.v2.3.9.jar mpileup2snp > samples.snp” command to detect SBSs. For InDels, using the “samtools mpileup -f ref.fa -q 10 samples_sorted.bam | java -jar VarScan.v2.3.9.jar mpileup2indel > samples.indel” command. Screening criteria were as follows: (1) the number of samples with an uncertain base at this site was zero (Samples NC = 0, the number of samples not covered or not called during sequencing). (2) The number of samples with genomic reference bases at this site was ≤ 1 (Samples Ref ≤ 1). (3) The allele frequency of reads supporting variations ≥ 30%, while < 10% in other samples (the variant allele frequency by read count). When the variant allele frequency of reading count was ≥ 75%, it was considered homozygous, refer to https://varscan.sourceforge.net/usinvarscan.html#v2.3_mpileup2snp for more details. Variants detected in non-irradiated parental lines and shared variants detected in different individual plants were removed. False positives in the detected mutations were counted and removed by Integrative Genomics Viewer (IGV) and Sanger sequencing (**Supplementary Table 2**) (Robinson et al., 2011). Then, the mutation sites were annotated. Based on the *O. sativa* Kitaake_499_V3.0 reference genome (Jain et al., 2019), SnpEff 5.0e (Cingolani et al., 2012) was used to annotate the detected variants and further analyze the effects of various mutation types on genes. The database was built from the Kitaake genome annotations. Further, we performed Gene Ontology (GO) analysis on the affected genes using eggNOG-mapper (eggNOG-mapper.embl.de) and TBtools (Chen et al., 2020). The biological process category was used in the GO analysis. We used eggNOG-mapper for functional annotation of Kitaake genes and to obtain background files for GO analysis. Then, TBtools (Chen et al., 2020) was used for enrichment analysis and plotting of the affected genes.

### 2.5 Mutant population construction, mutant screening, and yield traits measurement

In the M_2_ generation, 1172 M_2_ lines (ten plants per line) from eight CIB doses (25 – 200 Gy) were planted with a row spacing of 15 × 20 cm in Wenjiang, Sichuan (30°70′N, 103°83′E) for mutant screening. In the M_3_ generation, 129 mutant lines were planted with the same planting setting as M_2_ generation in Lingshui, Hainan (18°50′N, 110°04′E). In the M_4_ generation, the candidate high-yield mutants (A25, A28 and A96) were planted in Wenjiang, Sichuan, and the remaining mutants were planted in Lanzhou, Gansu (36°03′ N, 103°40′ E).

At full maturity, five plants were randomly selected from the middle of the row for measurement of plant height, panicles per plant, grains per panicle, and 1,000-grain weight. Plant height was measured in paddy fields. After the plump grains were dried in an oven at 42 °C for one week, the 1,000-grain weight was measured using the SC-A grain analysis system (Wanshen Ltd., Hangzhou, China). The yields of A25, A28 and A96 were measured and calculated per square meter (m^2^), with each mutant containing three independent replications.

### 2.6 Statistical analysis

Three replications were used for each treatment, and the data were presented as mean ± standard deviation (mean ± SD). The ANOVA analysis followed by *post-hoc* Tukey HSD was performed for significant differences (*P* < 0.05) using SPSS 29. Graphics were created using GraphPad Prism 9 and TBtools (Chen et al., 2020).

## 3 Results

### 3.1 Different CIB irradiation doses have different biological effects on the life cycle of M_1_ plants

**Figure 1:**
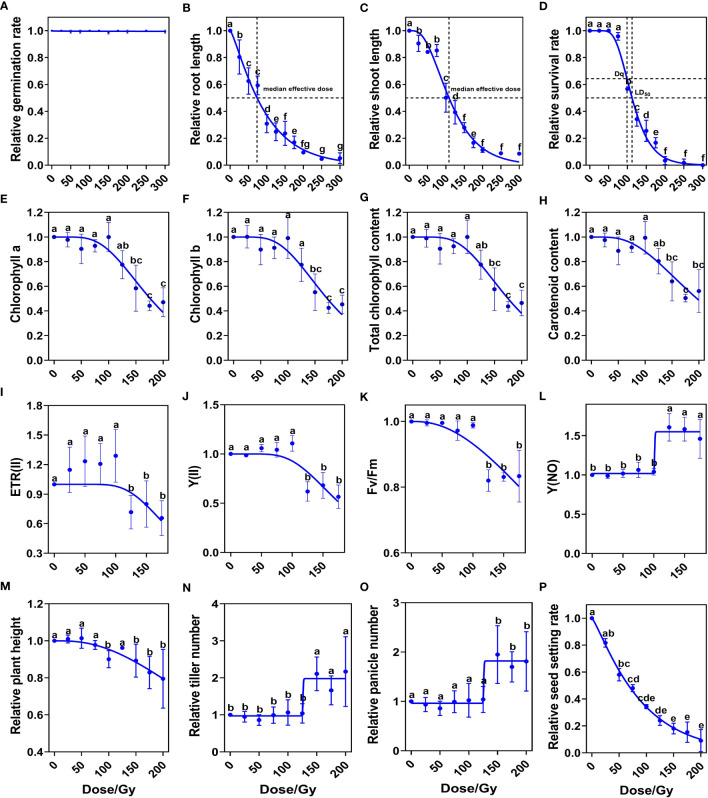
Biological response of M_1_ plants to different doses of CIB irradiation. **(A–D)** Relative values of developmental index at seedling stage. **(E–H)** Relative values of photosynthetic pigment contents at tillering stage. **(I–L)** Relative values of chlorophyll fluorescence parameters at tillering stage. **(M–P)** Relative values of indicators at maturity period. Data represent the mean ± SD of three replicates. Different lowercase letters represent significant differences among treatments (*P*<0.05) by ANOVA analysis. The single-hit multi-target (SHMT) model equation is S = 1 − (1 − EXP(−D/D_0_))^N^. **(A–K)**, **(M, P)** were fitted by nonlinear curves based on a SHMT model. **(L, N, O)** were fitted by nonlinear curves based on a dose-response-stimulation model.

We first systematically studied the biological effects induced by ten CIB doses (25, 50, 75, 100, 125, 150, 175, 200, 250 and 300 Gy). We found that seed germination (the emergence of both primary leaves and radicles) rate was over 98% at each CIB dose (**Figure 1A**). However, root lengths and shoot lengths of 9-day seedlings were significantly lower than control, even those in the 25 Gy group (**Figures 1B, C**). Using the single-hit multi-target (SHMT) model (Yoshitaka et al., 2019), we observed that the dose for reducing the shoot length to half of the control was 108.49 Gy, while the dose for reducing seedling root length to half of the control was 73.52 Gy. Thus, roots were more sensitive to CIB irradiation than shoots. Similarly, according to the SHMT model, the shoulder dose (Dq) of Kitaake was 98.82 Gy and the LD_50_ was 112.30 Gy. When the CIB dose was over 75 Gy, the survival rate dropped dramatically; almost all seedlings died at high irradiation doses (> 175 Gy) (**Figure 1D**).

We further investigated the effects of different CIB doses on chlorophyll contents and chlorophyll fluorescence parameters at tillering stage (30-day old). The contents of chlorophyll a, chlorophyll b, total chlorophyll, and carotenoid in leaves were significantly reduced when the irradiation dose was over 125 Gy (**Figures 1E–H**). These results indicate that high dose irradiation damages the chlorophyll biosynthesis pathway in rice. Chlorophyll fluorescence parameters showed that when the dose was lower than 100 Gy, the ETR (II), Y(II), Fv/Fm, and Y(NO) were almost unaffected. CIB greater than 100 Gy reduced ETR(II), Y(II) and Fv/Fm values (**Figures 1I–K**), but increased the Y (NO) value (**Figure 1L**). These results suggest that CIB irradiation above 100 Gy, due to irradiation damage, has a significant effect on photosynthesis at tillering stage in M_1_ plants.

We then measured agronomic traits at maturity, including plant height, tiller number, panicle number, and seed setting rate. We found no significant differences between treated groups and the control in plant height, tiller number, or panicle number, when CIB was lower than 125 Gy. However, when CIB was higher than 125 Gy, plant height decreased significantly (**Figure 1M**), and the numbers of tillers and panicles of survived plants of high-dose groups were significantly higher than those of control and low-dose groups (**Figures 1N, O**). Seed setting rate gradually decreased with increasing irradiation dose; at doses greater than 125 Gy, the seed setting rate dropped to less than 25% (**Figure 1P**). Therefore, we conclude that doses over 125 Gy cause serious damage on rice and have significant effects on normal development in the M_1_ generation.

### 3.2 CIB-induced genomic mutation frequency has obvious dose distribution intervals in M_2_ plants

**Figure 2:**
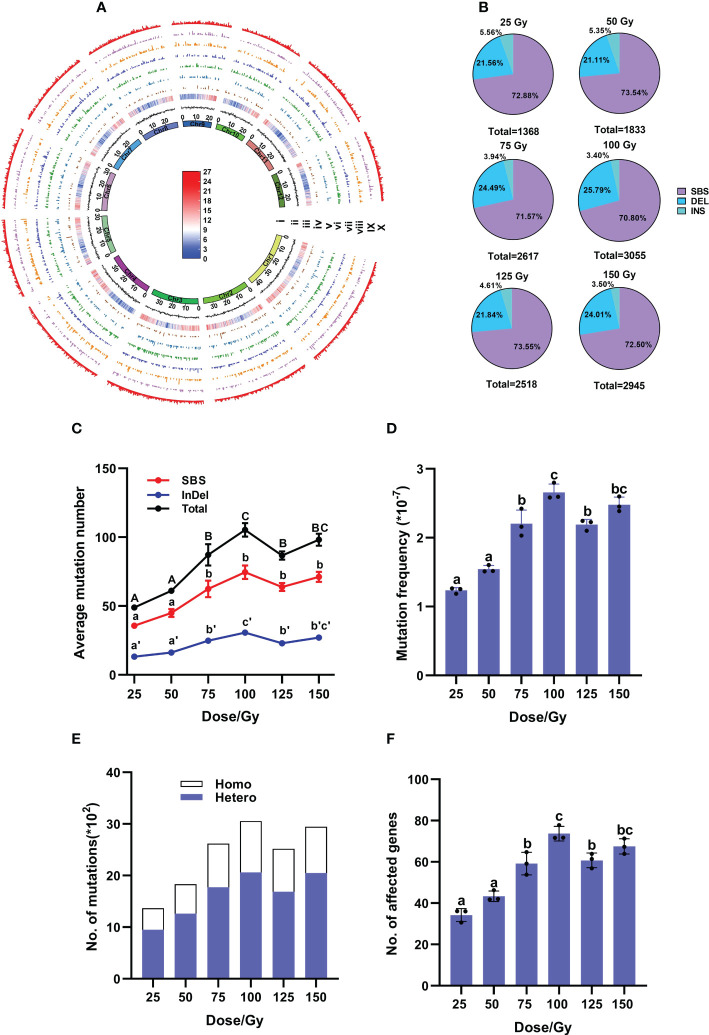
Genomic mutations induced by CIB at each dose in M_2_ population. **(A)** Genome-wide distribution of mutations induced by six CIB doses in M_2_ population. The circular tracks (from inner to outer) represent the 12 rice chromosomes on a megabase scale; GC ratio and gene density are on 100-kb windows of the reference genome KitaakeX; Tracks iv to X represent the mutations induced by doses of 25, 50, 75, 100, 125, and 150 Gy, as well as the number of all mutations induced by six doses of CIB within a non-overlapping 100-kb range of chromosomes. The highest signal heights in these tracks represent nine mutations/100-kb, seven mutations/100-kb, seven mutations/100-kb, nine mutations/100-kb, eight mutations/100-kb, nine mutations/100-kb, and nine mutations/100-kb. The color bar represents gene density within a 100-kb chromosome region, with red indicating an increase in gene density and the highest point representing 27 genes/100-kb. Blue represents a decrease in gene density, with the lowest point representing zero genes/100-kb. **(B)** Percentages of SBSs, deletions and insertions in each dose group. **(C)** Average number of induced mutations. **(D)** Mutation frequency at each dose. **(E)** Zygosity of induced mutations. **(F)** Average number of affected genes. **(C, D, F)** data represent the mean ± SD of three replicates. Different lowercase letters represent significant differences among treatments (*P*<0.05) by ANOVA analysis.

According to the biological effects in M_1_ plants after different doses of irradiation, we selected M_2_ samples from six CIB dose groups to further study the molecular characteristics of induced mutations. A total of 176 M_2_ lines and three control samples were re-sequenced (**Supplementary Table 1**). A total of 14,336 mutations, including 10,368 SBSs, 3,368 deletions and 600 insertions, were detected in the 176 samples of the six CIB-dose groups. We found that the mutations induced by CIB were unevenly distributed throughout the genome, and the number of mutations appeared not related to the sequencing depth or gene density (**Figure 2A**; **Supplementary Table 3**). We found that most mutations occurred in intergenic regions (30%) and upstream and downstream regions (25%), followed by introns (10%), exons (5%), and the 3’- and 5’-untranslated regions (UTR, 3%) (**Supplementary Figure 1**).

Single base substitutions (SBSs) were the most abundant mutations in each group, accounting for more than 70%, followed by deletions (21 – 26%) and insertions (about 5%) (**Figure 2B**; **Supplementary Table 4**). The ratios of SBSs/InDels had no significant differences among the six groups, ranging from 2.41 to 2.78 (**Figure 2B**; **Supplementary Table 4**). The number of induced mutations gradually increased from 25 to 100 Gy, but remained steady from 100 to 150 Gy, then even slightly decreased at 125 Gy, indicating that a saturated mutation level was reached at approximately 100 Gy. At 100 Gy, the average mutation number was 105.23 (± 4.00)/line and average mutation frequency was 2.66 (± 0.10) × 10^-7^/bp (Average number of mutations/Kitaake reference genome size), showing no significant differences from those (98.17 (± 4.40)/line and 2.48 (± 0.11) × 10^-7^/bp) at 150 Gy (**Figures 2C, D**). When zygosity of mutations was investigated, the ratio of heterozygous to homozygous sites at each dose was 2.02 – 2.30 (**Figure 2E**).

**Figure 3:**
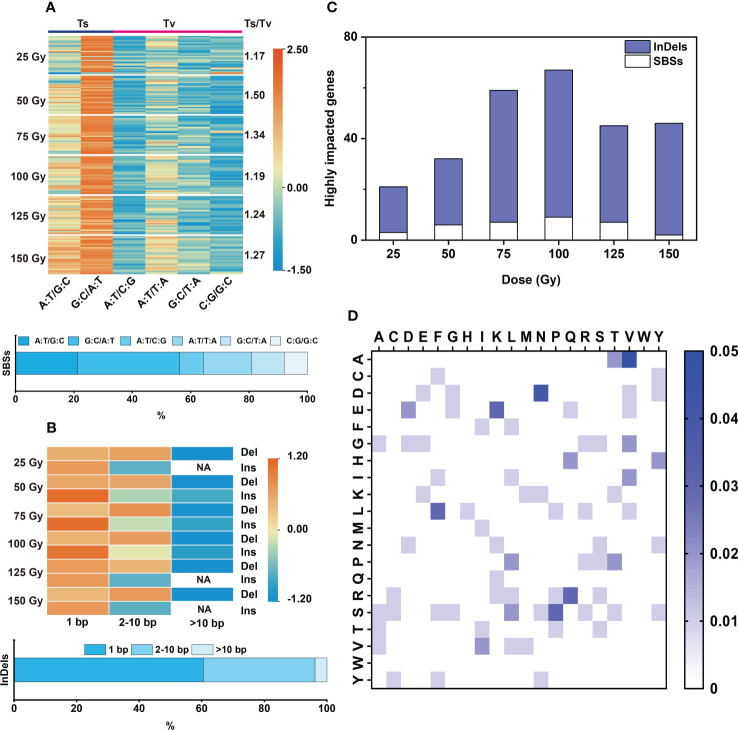
Characteristic of SBSs and InDels induced by each dose of CIB. **(A)** Type and proportion of SBSs, the color bar values represent the normalized using data row scale. **(B)** Size distribution of InDels, the color bar values represent the normalized using data row scale. NA represents no such mutation found. **(C)** Genes highly impacted by SBSs and InDels. **(D)** Heatmap of all amino acid variations induced by CIB. The color bar represents the frequency of the amino acid variation, with darker shades of blue indicating a higher frequency of the amino acid variation. The highest mutation frequency is 5%.

In the 176 individual M_2_ lines, 9,961 genes were affected, among them 253 genes were highly affected (including nonsense and frameshift mutations), 630 genes were moderately affected (missense mutations), 288 genes were only lightly affected (synonymous mutations), and 8,790 genes were in introns or intergenic regions (See http://snpeff.sourceforge.net for details) (**Supplementary Tables 5**, **6**). Among these affected genes, 2,899 were caused by InDels (29.1%) and 7062 caused by SBSs (70.9%) (**Supplementary Table 5**). Similarly, 100 Gy yielded the largest number of affected genes with an average of 74 (± 2.06)/line, including three highly-affected genes per line (**Figure 2F**; **Supplementary Table 6**). The highly affected genes were mostly caused by InDels (> 80%), only few (< 20%) by SBSs (**Figure 3C**).

For specific biological processes (Ashburner et al., 2000), we found from GO analysis that the most enrichment of all the genes affected by the six doses of CIB irradiation group was observed in polyacetaldehyde metabolism, polysaccharide decomposition metabolism, and cellulose decomposition metabolism(**Supplementary Figure 2**). Further GO analysis of different CIB irradiation group showed that they were enriched in different biological processes. The regulation of photoperiodism, flowering, cell tip expansion, and pollen tube growth had more hits at 25 Gy group, whereas the oligosaccharide catabolic process, the disaccharide catabolic process, and the carbohydrate catabolic process had more hits at 100 Gy group (**Supplementary Figure 2**). However, there were also some shared biological processes between different radiation dose groups. For example, there were 12 shared GO terms between the 75 Gy and 100 Gy radiation dose groups, including carbohydrate transport, plant epidermis morphogenesis, and positive regulation of cell differentiation, etc (**Supplementary Figure 3**; **Supplementary Table 7**). However, these biological processes were not among the top ten GO terms with the highest enrichment levels (the ten GO terms with the lowest *P*-values), and no consistent pattern was observed for the specific shared GO terms and genes for different doses group. Therefore, just like the uneven distribution of induced SBSs and InDels, the genes affected by CIB are also random.

### 3.3 CIB induces high rates of base-transitions and small InDels (<10 bp) in M_2_ generation

Transitions (Ts) and Transversions (Tv) are two forms of SBS. In this study, we found that the ratios of Ts/Tv induced by the six CIB doses were between 1.17 and 1.50. Therefore, CIB induced more Ts than Tv in M_2_ rice. Among the SBSs, G:C to A:T was the most abundant one (31.1 – 37.6%), followed by A:T to G:C (16 – 24.8%) and A:T to T:A (15.1 – 18.5%); A:T to C:G, G:C to T:A and C:G to G:C were the least (**Figure 3A**).

For each CIB dose, most of the induced InDels were deletions (more than 80%) (**Supplementary Table 4**). The induced deletions ranged from 1 to 38 bp and insertions ranged from 1 to 17 bp. The induced deletions were mainly 1 bp (45.16% on average) and 2-10 bp (48.03% on average), with few >10 bp deletions (6.81% on average). The induced insertions were mainly 1 bp (75.75% on average), with some 2-10 bp (23.43% on average) and very few >10 bp (0.82% on average) (**Figure 3B**).

### 3.4 Different CIB doses induce different amino acid variations in M_2_ plants

In the six CIB irradiation groups, 131 amino acid variants were induced, accounting for 34.5% of all possible amino acid variations (**Figure 3D**). In the 25 Gy group, only 36 amino acid variations were induced, accounting for only about 9.5% of possible variations. More than 60 amino acid variants were induced at 75 Gy, 100 Gy, and 150 Gy, accounting for roughly 16% of possible variations (**Supplementary Figure 4**). The wide distribution and large number of amino acid changes induced by CIB suggests that mutations induced by CIB are relatively random. The most abundant amino acid changes are as follows: proline to serine (12%, in 25 Gy group), aspartate to tyrosine (7%, 50 Gy group), lysine to glutamate (7%, 75 Gy group), arginine to leucine (6%, 100 Gy group), leucine to serine (8%, 125 Gy group), and phenylalanine to leucine (9%, 150 Gy group) (**Supplementary Figure 4**).

### 3.5 Few mutations induced by CIB are shared by different panicles of the same M_1_ plant

**Figure 4:**
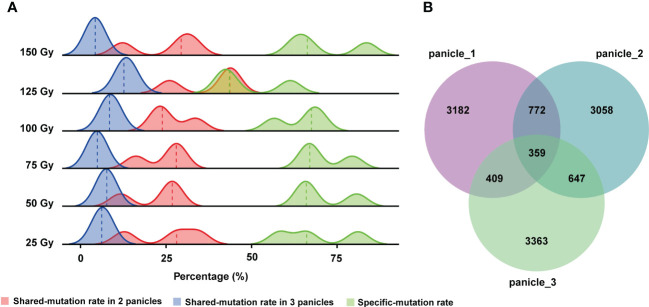
Rates of mutation unique to each panicle or shared by different panicles of the same M_1_ plant. **(A)** Rates of shared and unique mutations at each CIB dose. **(B)** Numbers of shared mutations and unique mutations in three panicles of a single M_1_ plant from all CIB groups.

Zheng et al. (2020) reported that after seeds were irradiated by HIB, different mutations were observed in different panicles of the same M_1_ plant. Hence, they speculated that different panicles might be derived from different progenitor cells, but it has not been verified at the molecular level (Zheng et al., 2020). Furthermore, the shared and unique mutation rates among different panicles from the same M_1_ plant were not reported in previous studies. Therefore, in this study, we counted the number of genomic mutations (SBSs and InDels) shared by different panicle from the same M_1_ rice plant. The results showed that the rate of mutations shared by the three re-sequenced panicles of a single M_1_ plant are very low, only about 7.5% on average, and the rate shared by two panicles was 20%. Consequently, the rate of unique mutations in a single panicle was very high averaging at 66.9%. Therefore, different panicles of a single M_1_ plant share few mutations and each panicle contains a large number of unique mutations (**Figure 4A**). Similar conclusions held true when all 176 lines were examined (**Figure 4B**).

### 3.6 CIB irradiation effectively generates many phenotypic mutants with a wide spectrum of mutations

**Figure 5:**
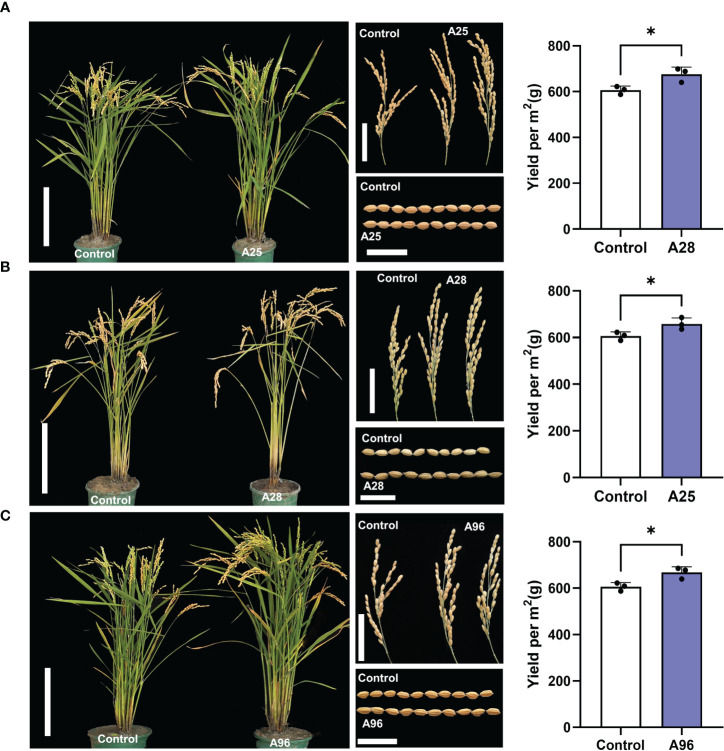
Three mutants showing higher yields. Morphologies of whole plant, panicle, and grain, and yields of mutants A25 **(A)**, A28 **(B)**, and A96 **(C)** and Kitaake control. Scale bars of plants = 20 cm, scale bars of panicles = 5 cm, and scale bars of grains = 1 cm. Yields were calculated per square meter (m^2^) containing 50 plants in rice paddy fields with three replications. The asterisk represents a significant difference (*P*<0.05) by Student’s T Test.

Because our goal for CIB-induced mutagenesis is to facilitate breeding, we next turned to agronomic traits. We found 129 mutants with diverse phenotypes from a population of 1,172 M_2_ lines (ten plants per line), accounting for a 1.1% rate. Among them, decreased seed setting was the most abundant one accounting for 49.6% of the phenotypic mutants, followed by plant height variations accounting for 23.3%. Among plant height variations, dwarf mutations accounted for 20.2% and increased plant height constituted 3.1%. We also obtained phenotypic mutants in grains (3.9%), tiller number (2.4%), plant architecture (4.7%), leaf (3.1%), panicle (4.7%) and growth period (6.2%) (**Figure 5**; **Supplementary Figures 5**, **6**; **Supplementary Table 8**). We also counted the numbers and mutation rates of phenotypic mutants in the M_2_ generation of all eight CIB doses (25 – 200 Gy) in the field. We found high phenotypic mutation rates (1.36 – 1.82%) at 75-150 Gy. Although a high dose (125 – 150 Gy) can also induce a high phenotypic mutation rate, it causes great damages including a low survival rate and low seed setting in M_1_ plants, rendering it difficult to obtain many seeds for further screening (**Supplementary Table 9**).

Among the 129 mutants, about 50% of the mutant phenotypes became no longer separable in the M_3_ generation, indicating that they are inherited as a qualitative trait. Ten mutants with more relevant agronomic traits were selected for further examination (**Figure 5**; **Supplementary Figure 6**; **Supplementary Table 10**). Among them, A36 displayed larger panicles but fewer tillers compared to control, and A45 showed larger seeds but lower seed setting, fewer tillers, and shorter plants (**Supplementary Figure 6A**; **Supplementary Table 10**). A50, A60, and A110 were dwarf plants, with smaller panicles and shorter grains (**Supplementary Figure 6B**; **Supplementary Table 10**). A66 showed longer panicles and beak-shaped grains (triangular hulls). A8 showed longer panicles with smaller grains (**Supplementary Figure 6C**; **Supplementary Table 10**). Most interestingly, we also found three mutants (A25, A28, and A96) showing enhanced yields that have potentials to serve as breeding resources. These three mutants yielded significantly more grains (by 8.5 – 11.5%) than the control, calculated per m^2^ containing 50 plants in rice paddy fields. A25 showed a taller plant stature, more tillers, and larger panicles resulting in higher grain yield (**Figure 5A**; **Supplementary Tables 10**, **11**). A28 carried a taller stature, longer panicles, and larger grains, also resulting in higher yields (**Figure 5B**; **Supplementary Tables 10**, **11**). A96 exhibited a taller stature, larger panicles and larger grains, leading to higher grain yields (**Figure 5C**; **Supplementary Tables 10**, **11**).

### 3.7 Mutation characteristics and candidate gene prediction of 11 phenotypically stable M_4_ mutants

**Figure 6:**
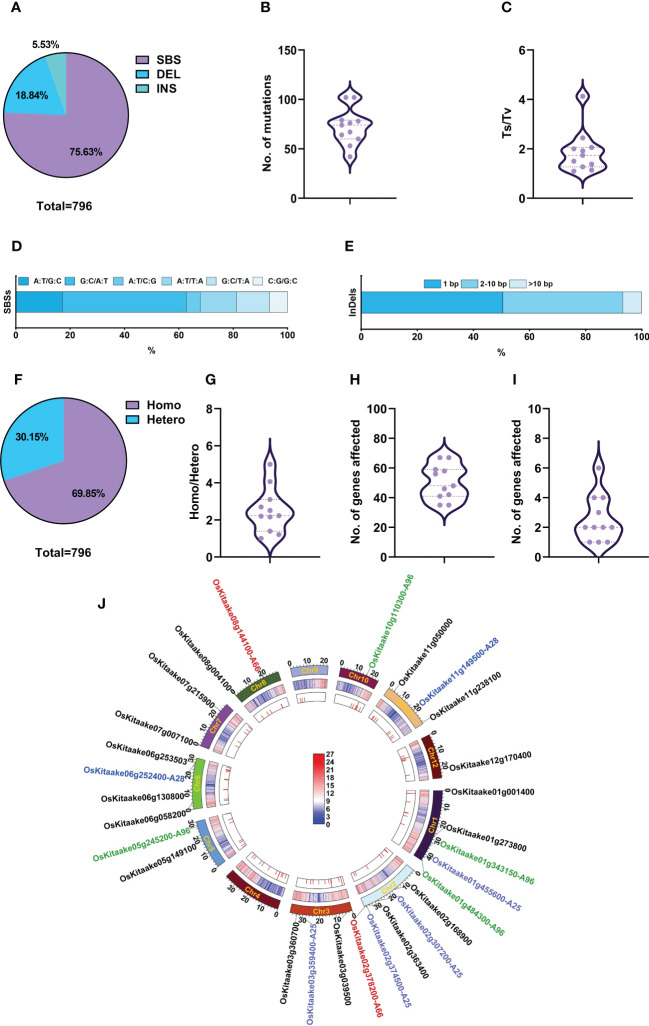
Genomic variations and candidate genes of phenotypically stable M_4_ mutants. **(A)** Percentage of SBSs, deletions, and insertions. **(B)** Number of mutations in each mutant line. **(C)** Ts/Tv value in each mutant. **(D)** Proportion of various types of SBSs. **(E)** Size distribution of InDels. **(F)** Proportion of homozygous and heterozygous mutations in M_4_ generation. **(G)** Zygosity of each mutant. **(H)** Total number of affected genes in each mutant. **(I)** Highly and moderately impacted genes in each mutant. **(J)** Circos diagrams of mutations in M_4_. From the outer track to the inner track: the 12 chromosomes of rice represented on a megabase scale with the candidate genes on them, gene density on 100-kb windows of reference genome KitaakeX, and distribution of mutations on each chromosome in 11 mutants. The color bar represents gene density within a 100-kb chromosome region, with red indicating an increase in gene density and the highest point representing 27 genes/100-kb. Blue represents a decrease in gene density, with the lowest point representing zero genes/100-kb. The colors of the candidate genes, A25, A28, A96 and A66 for the mutants were highlighted with the colors being purple, green, red, and blue respectively. The candidate genes for the other mutants were displayed in black.

In the M_4_ generation, we selected 11 mutants with stable mutant phenotypes, including A18, A90, and the above-described nine mutants with agronomic traits, and re-sequenced them to identify induced SBSs and small InDels. A total of 796 mutations were detected, including 602 SBSs (75.6%), 150 deletions (18.8%), and 44 insertions (5.5%) (**Figure 6A**). The average number of mutations in each mutant was 72.3 (54.7 SBSs + 17.6 InDels), with the minimal number in A18 at 42 (35 SBSs + 7 InDels) and the maximal number in A96 and A110 at 102 (75 SBSs + 27 InDels) (**Figure 6B**; **Supplementary Table 12**). The Ts/Tv ratio of SBSs was 1.1 – 4.1, with an average of 1.88 (**Figures 6C, D**). Among the SBSs, G:C to A:T was also the most abundant one (45.7%), followed by A:T to G:C (17.1%), A:T to T:A (13.3%) and G:C to T:A(12.1%); A:T to C:G and C:G to G:C were the least (about 5%). Deletions accounted for 77.7% of InDels, and the ratio of 1 bp: 2-10 bp: >10 bp deletions was 5: 5.54: 1; the ratio of 1 bp: 2-10 bp insertions was 3: 1 (**Figure 6E**; **Supplementary Tables 12**, **13**). The ratios of homozygous/heterozygous mutations were 1 – 5, with an average of 2.51 (**Figures 6F, G**; **Supplementary Tables 12**, **13**).

Like the M_2_ generation, M_4_ mutants did not show a special pattern in the distribution of mutations (**Figure 6J**). The locations of mutations showed that ~70% of mutations (554) were in genic regions and 30% in intergenic regions (**Supplementary Figure 1**). In total, 796 mutations affected 554 genes, including 14 highly affected, 29 moderately affected, 12 lightly affected and 499 unaffected genes. Each mutant contains 41 – 67 affected genes (**Figures 6H, I**; **Supplementary Table 14**). As these 11 M_4_ lines were phenotypically stable, we only regarded homozygous mutations that highly or moderately impacted genes as candidate genes. Therefore, we selected 27 candidate genes for the 11 mutants, with an average of 2.46 candidates per line (**Figures 6I, J**; **Supplementary Table 14**). Their gene functions were revealed by searching the Kitaake eggNOG-mapper library and Rice Genome Annotation Project (http://rice.uga.edu/analyses_search_locus.shtml). For example, we selected two candidate genes, *OsKitaake02g378200* (*LOC_Os02g56610*) and *OsKitaake08g144100* (*LOC_Os08g30810*) (**Figure 6J**; **Supplementary Table 14**), for A66 which is a mutant showing beak-shaped grains or triangular glumes (**Supplementary Figure 6C**). Previous studies have shown that *OsKitaake02g378200* (*LOC_Os02g56610*) encodes a DUF640 domain protein, which controls the development of rice lemma and palea. Mutations in this gene caused beak-shaped grains or triangular glumes (Li et al., 2012; Ma et al., 2012; Wei et al., 2013; Yan et al., 2013; Ren et al., 2016), which is very similar to A66 phenotypes. The other candidate gene *OsKitaake08g144100* (*LOC_Os08g30810*) encoding a puromycin-sensitive aminopeptidase had no available phenotypes. Therefore, we attribute the phenotype of A66 to *OsKitaake02g378200* (*LOC_Os02g56610*) on chromosome 2 with a G deletion at position 35911065, causing a frameshift. The candidate genes and description of functions for other mutants are listed in **Figure 6J** and **Supplementary Table 14**.

## 4 Discussion

### 4.1 Systematical analysis of the effects of different CIB doses on growth phenotypes in M_1_ rice to identify an optimal dosage

Previous studies on the biological effects in M_1_ generation induced by HIB irradiation were mostly partial. For example, the irradiation dose used and the measured indicators mostly only included survival rate and seed setting rate (Hase et al., 2018; Zheng et al., 2020), which may lead to inaccurate selection of optimal irradiation doses. Here, we systematically investigated the biological effects induced by a wide range of dosage (10-CIB-dose) for the whole life cycle of M_1_ generation, including growth, development, and photosynthetic indicators. Therefore, we greatly supplemented and improved the approach to more completely examine the biological effects induced by different CIB doses in rice. Based on the growth, development and photosynthetic observations on M_1_ generation, we conclude that the upper limit of CIB doses to induce mutations for breeding Kitaake rice is ~125 Gy (**Figures 1A–P**).

### 4.2 Quantity and types of mutations induced by different doses of CIB in M_2_ generation

The M_2_ individuals treated with six CIB doses (25 – 150 Gy) were further used to study the types of genomic mutations. Here, we randomly selected a large number of M_2_ samples (176 in total, about 30 for each dose group) rather than a small number of samples with observable phenotypes in higher generations (M_3_ - M_6_) as used in most previous studies (Du et al., 2017; Li et al., 2019; Yang et al., 2019; Zheng et al., 2020), which were obviously biased. Therefore, our research on the mutagenic characteristics of CIB should obtain more accurate numbers. Only SBSs and InDels induced by CIB with different doses were investigated here because of two reasons. First, previous studies have shown that HIB with low LET mainly induces SBSs and small InDels (Kazama et al., 2013; Kazama et al., 2017). Second, due to the limitation in the short read length (2×150 bp) of the next generation sequencing (NGS), the false positive rate of structural variation (SV) detection is quite high. We found that, in the M_2_ generation, SBSs were the most abundant type of genomic variations for each dose of CIB, accounting for more than 70%, and InDels accounted for less than 30% (**Figure 2B**). The number of mutations increased gradually from 25 to 100 Gy CIB, but remained steady from 100 to 150 Gy, showing a peak value at 100 Gy (**Figure 2C**; **Supplementary Tables 4**, **5**). Meanwhile, 100 Gy CIB treatment affected the highest number of genes at 74 genes per plant (with 6.4 highly or moderately affected), but still far less genes than other mutagenic methods, such as EMS (Thompson et al., 2013).

### 4.3 The optimal CIB dose range for construction of mutant populations and breeding of rice

Consequently, based on the biological effects of different CIB doses on M_1_ plants, the WGS results of M_2_ lines, and the field results of M_2_ generation from eight CIB doses, we conclude that the optimal dosage for rice mutagenesis by CIB is 75 – 100 Gy, which causes 75 – 100% Dq or 67 – 90% LD_50_. At this dose range, the M_1_ population has relatively low damages, retaining normal growth, development, and photosynthetic physiology, with a moderate survival rate and fertility (**Figures 1A–P**). With this dosage, more genomic mutations were generated in the M_2_ generation (**Figures 2A–C**), and more mutant lines can be screened in the field, leading to a higher rate of phenotypic variations. As the response to CIB may vary with genetic backgrounds (varieties), the CIB dose-setting can be to be adjusted within a range for different rice varieties or even different crops in practical application.

### 4.4 Differences of mutation characteristics in different genetic generations of rice

We selected 11 M_4_ stable mutants to explore their mutation characteristics. We found that there were some differences in mutation characteristics between M_2_ and M_4_ samples. For example, the proportions of SBSs (75.6%) and insertions (5.5%) in M_4_ (**Figures 2B**, **6A**) was lower than in M_2_ (23.16%) (**Figures 2B**, **6A**). Deletions accounted for about 77.7% of InDels in M_4_ generation (84.88% M_2_); the ratio of 1 bp to 2-10 bp to >10 bp at 5: 5.54: 1 in M_4_ was significantly different from the 15: 9: 1 ratio in M_2_, but the 1 bp to 2-10 bp insertion ratio of 3:1 in M_4_ was not significantly different from the 2.73: 1 ratio in M_2_ (**Figures 3B**, **6E**).

The Ts/Tv in each M_4_ line fluctuated widely, ranging from 1.1 to 4.1 with an average of 1.88, while the M_2_ Ts/Tv rates were between 1.17 and 1.50, with an average of 1.28 (**Figures 3A**, **6C, D**). Therefore, deviations may occur using higher-generation populations and a small number of mutants to evaluate Ts and Tv. Another obvious difference is that the proportion of homozygous mutations of the 11 M_4_ mutants was significantly higher, with the homozygous/heterozygous ratio falling between 1 and 5 and averaging at 2.51. However, this proportion in the M_2_ generation only showed an average of 0.429 (**Figures 2E**, **6F**). The average mutation frequency of the M_4_ mutants (1.83 × 10^-7^/bp) was similar to that of the M_2_ mutants, indicating that most of the induced SBSs and small InDels were heritable (**Figure 2D**). The locations of mutations in the genome in M_4_ and M_2_ generations were not significantly different (**Figures 2A**, ** 6J**). Therefore, the spectrum of genomic variations is related to the selected generations.

### 4.5 Comparison of genomic variations induced by CIB and other mutagens

Various physical and chemical mutagens have been applied to mutational breeding. Mutation characteristics induced by different mutagens are distinct. For instance, EMS, the most widely used chemical mutagen, mainly induces point mutations evenly distributed in the genome. The mutation frequency induced by EMS is profoundly high (~1.18×10^-6^/bp), with G:C to A:T change being the most abundant mutation accounting for ~88% (Thompson et al., 2013; Henry et al., 2014; Li et al., 2017). Although SBSs were also the most abundant mutation type induced by CIB, it was not evenly distributed in the genome in our case (**Figure 2A**). CIB can also induce multiple-base mutations, such as small InDels (**Figures 2B**, **3B**). InDels are more likely to induce nonsense and frameshift mutations. In this study, about 87% of highly affected genes in M_2_ are caused by small InDels (**Figure 3C**; **Supplementary Table 6**). Although G:C to A:T changes were the most abundant SBSs induced by CIB, its proportion was less than 40% (**Figure 3A**). Hence, the SBS variations induced by CIB were less biased, and more types of amino acid changes were induced (**Figure 3D**; **Supplementary Figure 4**).

The fact that the number of affected genes in each line was less (**Figures 2F**, **6H, I**) can be another advantage for CIB because this means only a small segregating population is needed to determine the causative mutation derived from CIB irradiation. γ-rays, the most widely used physical mutagen, mainly induces SBSs and small InDels, with SBSs accounting for ~70% in M_3_ - M_6_ generation (Li et al., 2019; Yang et al., 2019; Du et al., 2021). Like CIB, the mutations induced by γ-rays are unevenly distributed in the genome and mainly occur in the intergenic, upstream, and downstream regions of the genome (Yang et al., 2019). The difference is that CIB can induce more InDels than γ-rays, especially InDels >5 bp and larger fragments. Larger InDels may cause more highly affected genes leading to more phenotypic variations (**Figure 3B**) (Yang et al., 2019). Moreover, for heavy ions, different ion types have different LET, and the ion beam irradiation with higher LET, such as argon ion, is more likely to induce larger InDels and SVs. Therefore, the ion type can be selected according to the purpose of mutagenesis (Kazama et al., 2017). SBSs and small InDels induced by FN, which is another high-LET physical mutagen, are significantly different from those induced by CIB. For example, although SBSs were also the main type induced by FN in the M_2_-M_3_ generations of rice, its proportion (48%) was significantly lower than by CIB (> 70%), and the proportion of InDels induced by FN (40%) was significantly higher than that by CIB (average 27.5%, in this study). Therefore, researchers can select appropriate mutagens according to their purposes.

### 4.6 CIB enriches genetic resources for rice functional genomics research

In this study, we studied the phenotypes of 11 M_4_ stable mutants in detail and predicted their causal genes based on WGS (**Figure 5**, **Supplementary Figures 5**, **6**; **Supplementary Table 14**). Since these mutant traits are stable in M_2_ - M_3_ generations, we reason that their mutant phenotypes might be controlled by homozygous mutations in single genes. *OsKitaake02g378200* (*LOC_Os02g56610*) and *OsKitaake08g144100* (*LOC_Os08g30810*) are predicted as the candidate genes for A66 which displays beak-shaped grains/triangular hulls (**Supplementary Figure 6C**; **Supplementary Table 14**). Previous studies have reported that *OsKitaake02g378200* (*LOC_Os02g56610*) mutation caused a similar phenotype (Li et al., 2012; Ma et al., 2012; Wei et al., 2013; Yan et al., 2013; Ren et al., 2016). Therefore, we attribute the phenotypic change of A66 to the G deletion at position 35911065 on chromosome 2 resulting in a frameshift in *OsKitaake02g378200* (*LOC_Os02g56610*). This also demonstrates that our prediction method is feasible. Of particular interest is A28, a mutant with good yield traits including taller plants, longer panicles, larger grains, that has two predicted candidates, *OsKitaake06g252400* (*LOC_Os06g46400*) and *OsKitaake11g149500*(*LOC_Os11g32270*) (**Figure 5B**; **Supplementary Table 14**). We found that *OsKitaake06g252400* is highly expressed in shoots, pistil, anther, embryo of 25-DAP (days after pollination), and panicles (with Fragments Per Kilobase of exon model per Million mapped fragments (FPKM) > 5.5), while *OsKitaake11g149500* is not expressed in shoots (See http://rice.uga.edu/cgi-bin/ORF_infopage.cgi for detail). Therefore, we reason that *OsKitaake06g252400* is the more likely gene that causes the A28 mutant phenotype. A25 and A96 also carry improved yields, and their causal genes can be further studied focusing on the predicted candidate genes (**Figures 5A, C**; **Supplementary Table 14**).

In this study, we identified 27 candidate genes in 11 mutants (**Supplementary Table 14**). We searched the functions of these genes in the Kitaake eggNOG-mapper library and Rice Genome Annotation Project (http://rice.uga.edu/analyses_search_locus.shtml), but the functions of six remain unknown: *OsKitaake03g360700* (*LOC_Os03g56724*) of A8 (a mutant of longer panicles with less panicles and smaller grains, **Supplementary Figure 6C**), *OsKitaake01g001400*(*LOC_Os01g01170*), *sKitaake01g343150* and *OsKitaake05g245200* (*LOC_Os05g46440*) of A18 (a dwarf mutant with low seed setting rate, **Supplementary Figure 5V**), *OsKitaake01g343150* and *OsKitaake05g245200* (*LOC_Os05g46440*) of A96 (a mutant of taller plants, larger panicles, and larger grains, **Figure 5C**), and *OsKitaake12g170400*(*LOC_Os12g36270*)of A50 (a dwarf mutant with smaller panicles and shorter grains, **Supplementary Figure 6B**). These genes are good candidates for further functional analysis. In conclusion, our research provides valuable new genetic resources for rice breeding.

### 4.7 CIB has a great mutagenic potential

In this study, we screened various rice materials with visible phenotypes in M_2_ generation, including variations in plant height, grains, panicles, fertility, stem and leaf, which showed a broad spectrum of phenotypic mutations induced by CIB. In M_3_ generation, many (49.6%) lines show stable inheritance, which further demonstrates that a relative short period is needed to stabilize these mutations for breeding. There were about 70% heterozygous mutations in the M_2_ generation (**Figure 2E**). These heterozygous sites may be further separated to produce new phenotypic mutants in the offspring. The mutants obtained in this study enrich germplasm resources for Kitaake and for rice breeding, especially, the mutants with enhanced yield traits can be directly used in breeding practice. These phenotypic mutants can also be used to explore specific genes or alleles controlling special phenotypes by forward genetics or reverse genetics (Li et al., 2017; Sun et al., 2022).

Previous research proposed a hypothesis that different panicles may originate from different progenitor cells (Zheng et al., 2020). However, it has not been verified at the molecular level. In this study, we re-sequenced plants from three different panicles derived from a single M_1_ plant and validated this hypothesis. Moreover, our WGS results showed that the proportion of mutations shared by different panicles from the same plant was relatively low (**Figure 4**). Therefore, different panicles of M_1_ may be separately collected and treated as lines of independent mutational origins, which would greatly increase the number of mutants in the population. In addition, we only screened for visually observable mutants in this study, but there are many other mutants, such as low cadmium absorption, disease resistance, salt and alkaline tolerance, higher grain quality, that would be very useful to researchers and breeders.

## 5 Conclusion

**Figure 7:**
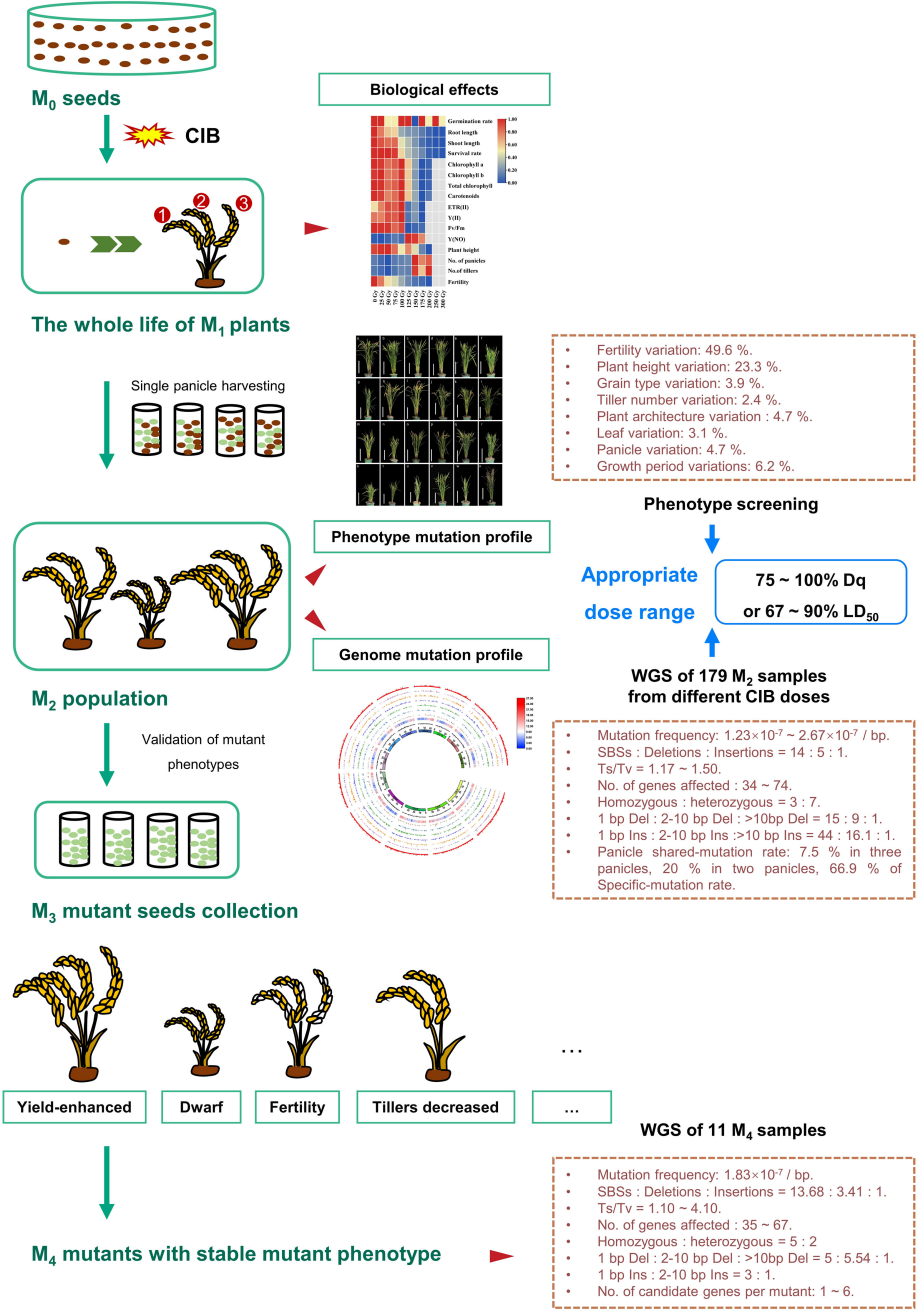
Overview of the mutagenic effects of different CIB doses on successive multi-generations of rice. First, after the Kitaake M_0_ seeds were irradiated by ten CIB doses (25 – 300 Gy), the biological effects of different doses in the whole life cycle of M_1_ generation were systematically studied. Second, each single panicle (M_2_ seeds) of the same plant was separately harvested at the mature stage. Furthermore, the genomic variations in M_2_ plants and mutations unique to a single panicle or shared by different panicles of the same M_1_ plant induced by six CIB doses (25 – 150 Gy) were studied. Third, we also constructed M_2_ mutant populations from eight CIB doses (25 – 200 Gy). By examining the biological effects in M_1_ generation induced by ten CIB doses, the mutation characteristics in M_2_ generation induced by six CIB doses, and the M_2_ phenotypic mutation rates induced by eight CIB doses, we conclude that the optimal dosage should cause 75 – 100% Dq or 67 – 90% LD_50_. In addition, we selected a variety of phenotypic mutants in the M_2_ mutant population and verified the isolated mutants in M_3_ - M_4_ generations. Finally, we selected 11 M_4_ phenotypic mutants to study their mutation characteristics and candidate genes.

This study comprehensively studied the mutagenic effects of different CIB doses in continuous multi-generations (M_1_ - M_4_) of Kitaake. We assessed the biological effects induced by different doses of CIB in M_1_ generation and the types of genomic variations induced by different doses of CIB in M_2_ generation. An M_2_ population induced by different CIB doses was constructed that contains a large number of valuable mutants. We estimated the optimal CIB dose range to be 75 – 100 Gy (75 – 100% Dq or 67 – 90% LD_50_) for Kitaake (**Figure 7**). In the M_4_ generation, the mutation types of stable phenotypic mutants were studied, and candidate genes for many mutants were predicted. The comparison between unselected M_2_ and M_4_ mutants revealed differences of mutations carried in different generations. Meanwhile, great mutagenic potential of HIB was also revealed: mutants from different panicles of a single M_1_ plant can be screened separately, and the induced heterozygous mutations can be screened in higher generations. We also obtained three high-yield mutant lines which can be used for rice breeding and genetic analysis of these agronomic traits.

## Data availability statement

The datasets presented in this study can be found in online repositories. The names of the repository/repositories and accession number(s) can be found below: https://ngdc.cncb.ac.cn/search/?dbId=&q=CRA010864, CRA010864.

## Author contributions

LZ, WR, YD and LL designed the experiments. WR, HW, YD, YL, ZF, GK, CX, XC, XL and CY carried out the experiments. LY and WJ performed the sample irradiation. HW, XZ, FY and JL carried out rice field planting. WR wrote the manuscript. YD, YL, LL, QS, TG, HG, JM and WL revised the manuscript. All authors contributed to the article and approved the submitted version.
