# Supplementary material for: Multi-generation study of heavy ion beam-induced mutations and agronomic trait variations to accelerate rice breeding
Source: Front Plant Sci. 2023 Jun 21;14:1213807. doi: 10.3389/fpls.2023.1213807 (PMC10322207; doi:10.3389/fpls.2023.1213807)
Supplement: Supplementary file 1 [file DataSheet_1.docx]

Supplementary Material

# Supplementary Figures

## Supplementary Figures

**Supplementary Figure 1.** Locations of mutations induced by each CIB dose. The upstream and downstream regions of a gene refer to the range of within 2-kb upstream and downstream of the gene, respectively.


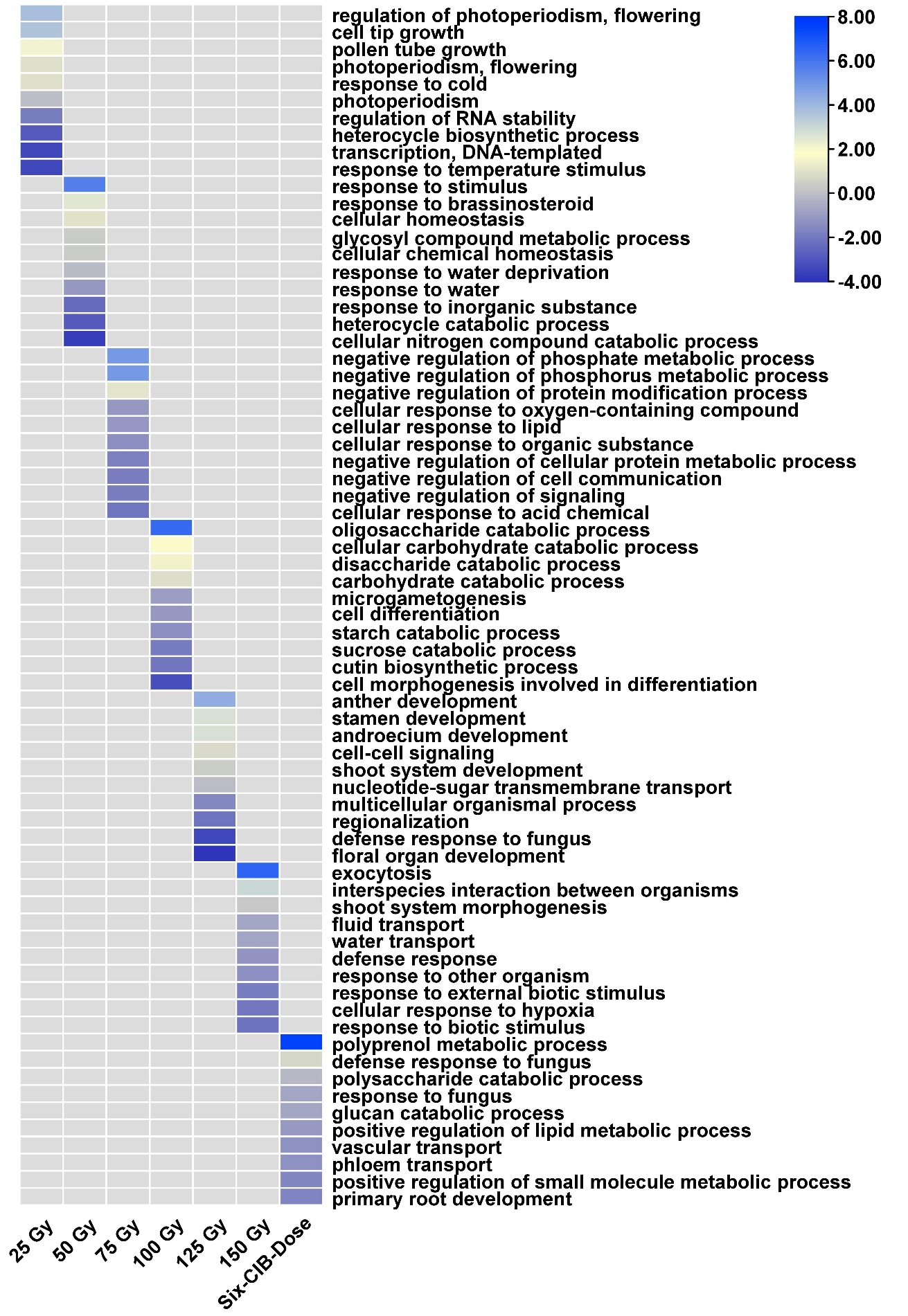
**Supplementary Figure 2.** GO enrichment analysis of genes affected by each of six CIB doses. The values on the color bar represent -log10(*P*-value) and were normalized according to the column.


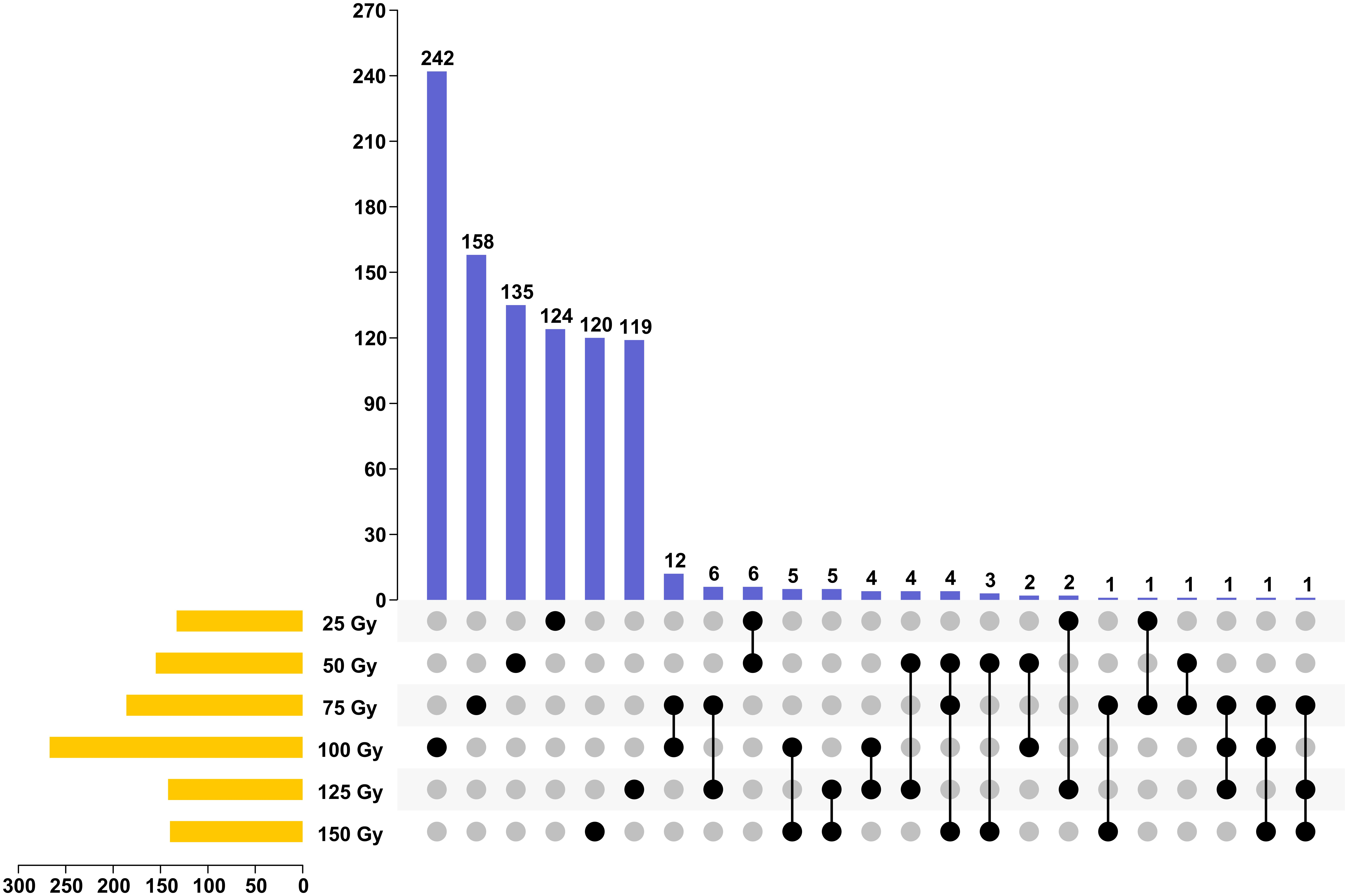


**Supplementary Figure 3.** Upset plot of GO terms affected by each of six CIB doses.

**Supplementary Figure 4.** Amino acid variations caused by SBSs induced by each CIB irradiation dose. Amino acid variations induced by 25 Gy **(A)**, 50 Gy **(B)**, 75 Gy **(C)**, 100 Gy **(D)**, 125 Gy **(E)**, and 150 Gy **(F)** CIB irradiation. The color bar represents the frequency of the amino acid variation, with darker shades of blue indicating a higher frequency of the amino acid variation. The highest frequencies from (A) to (F), were 12%, 7%, 7%, 6%, 8%, and 9% respectively.


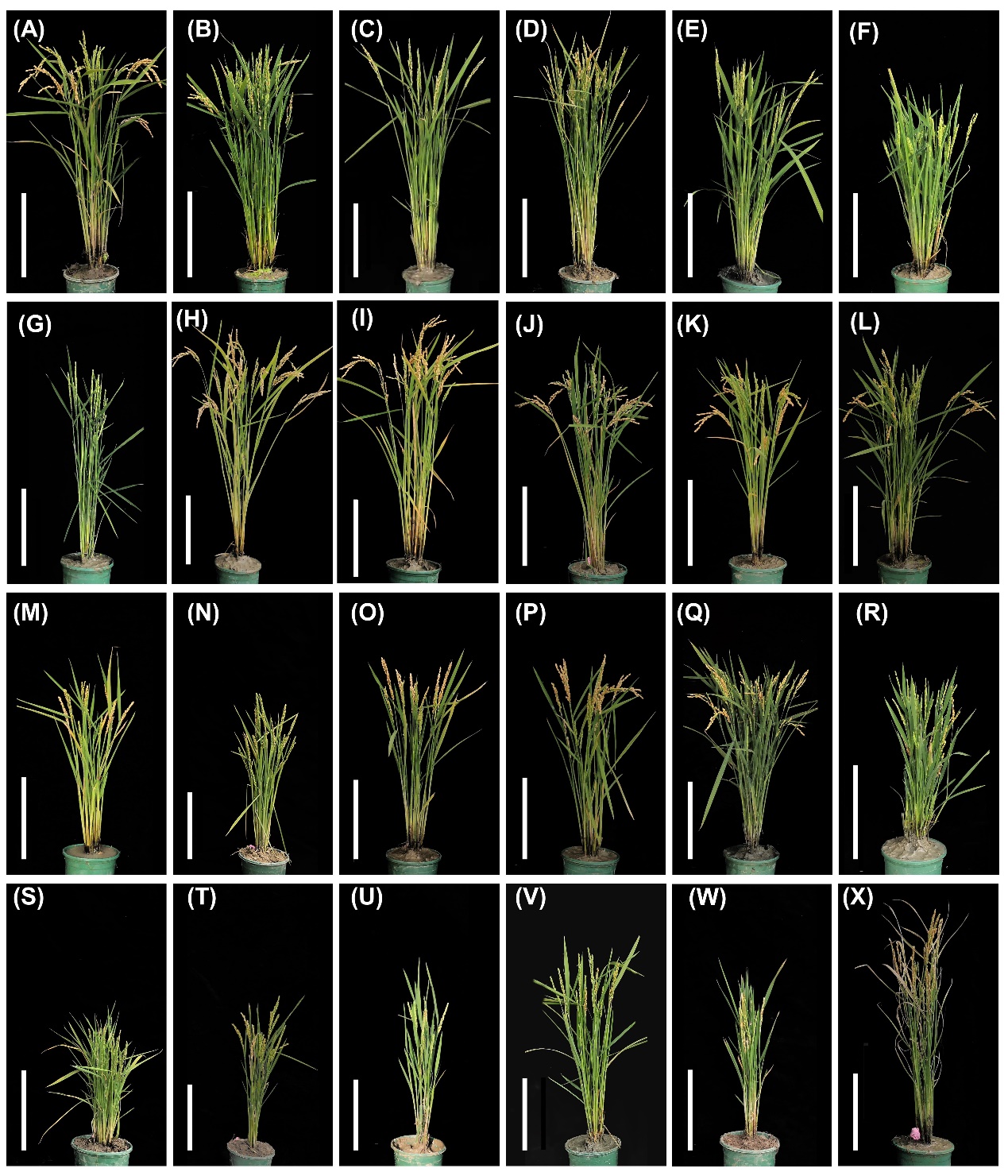


**Supplementary Figure 5.** Mutants of various phenotypes in M_3_ generation. **(A)** Control plant. **(B)** - **(D)** Lower seed setting. **(E)** Dwarf, lower seed setting, awned. **(F)** Dwarf, more tillers, smaller panicles, and lower seed setting rate. **(G)** Dwarf, narrow leaves, lower seed setting. **(H)** - **(I)** Taller plants. **(J)** - **(W)** Dwarf to different levels. **(X)** Early senescence.


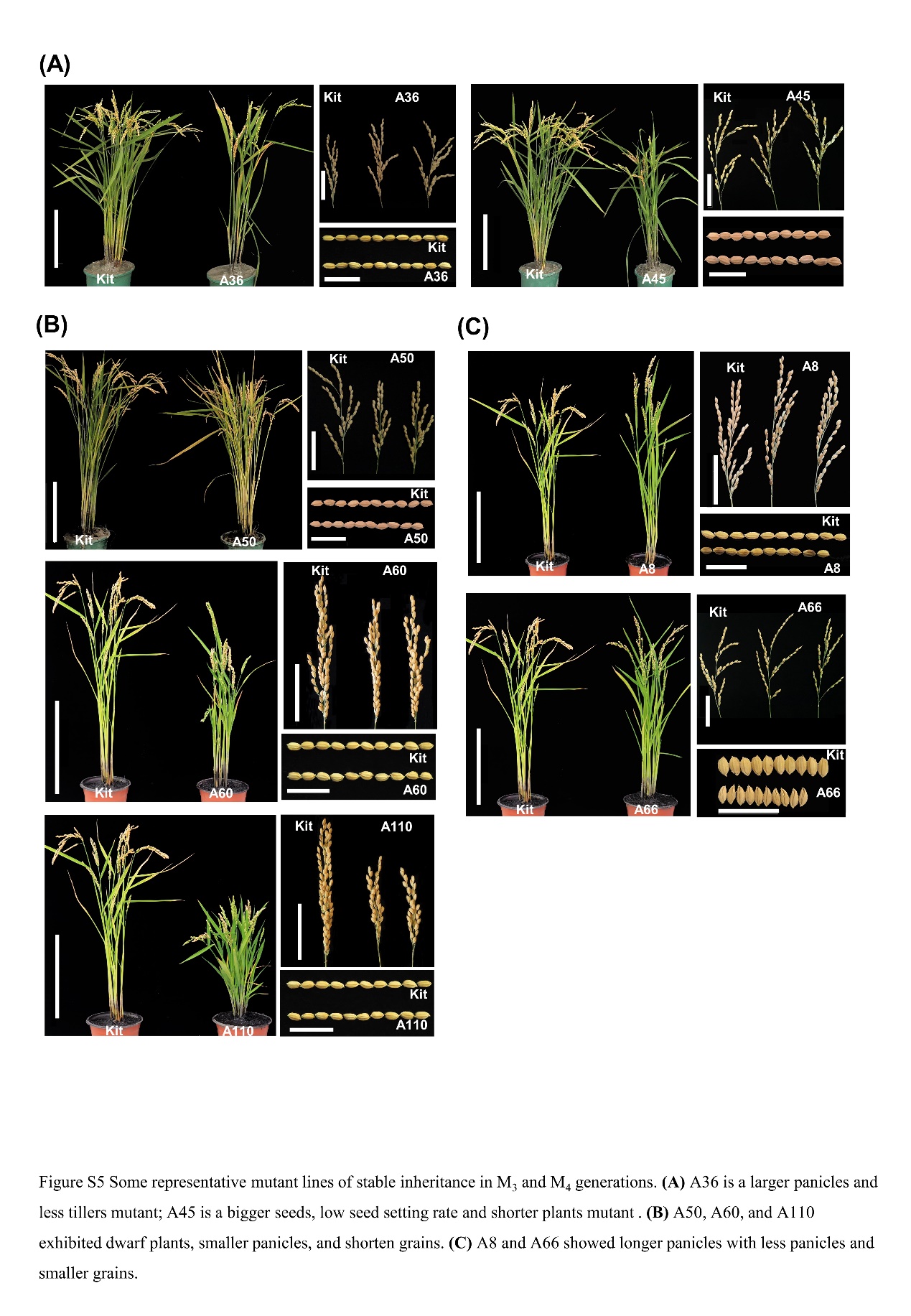


**Supplementary Figure 6.** Some representative mutant phenotypes stably inherited to M_3_ and M_4_ generations. **(A)** A36 has larger panicles and fewer tillers; A45 has bigger seeds, lower seed setting rate, and shorter plants. **(B)** A50, A60, and A110 exhibit dwarf plants, smaller panicles, and shorter grains. **(C)** A8 and A66 show longer panicles with smaller grains.
